# An Overview on Carbon Fiber-Reinforced Epoxy Composites: Effect of Graphene Oxide Incorporation on Composites Performance

**DOI:** 10.3390/polym14081548

**Published:** 2022-04-11

**Authors:** Harsh Sharma, Ajay Kumar, Sravendra Rana, Liberata Guadagno

**Affiliations:** 1 School of Engineering, University of Petroleum and Energy Studies (UPES), Dehradun 248007, Uttarakhand, India; engharsh.5@gmail.com; 2 Department of Industrial Engineering, University of Salerno, Via Giovanni Paolo II 132, 84084 Fisciano, Italy

**Keywords:** carbon fibers, graphene oxide, fiber-reinforced composites, interfacial adhesion, functional groups, mechanical performance

## Abstract

Carbon fiber-reinforced polymer (CFRP) composites are used in a variety of applications such as aircraft, automobiles, body armors, and the sports sector owing to their ultra-strong and lightweight characteristics. However, the incorporation of an untreated pristine carbon fiber surface leads to a weak interfacial interaction with the polymeric matrix, thus triggering catastrophic failure of the composite material. Graphene oxide, a 2D-macromolecule consisting of several polar functional groups such as hydroxyl, carboxyl, and carbonyl on the basal planes and edges, tends to increase the surface area and has thus been applied between the fiber and matrix, helping to improve CFRP properties. Herein, we condense different routes of functionalization of GO nanosheets and their incorporation onto a fiber surface or in a carbon fiber-reinforced epoxy matrix, helping to improve the interfacial adhesion between the fiber and matrix, and thus allowing effective stress transfer and energy absorption. The improvement of the interfacial adhesion between the fiber and carbon fiber-reinforced epoxy matrix is due to the peculiar structure of GO nanoparticles composed of polar groups, especially on the edges of the nanosheets, able to provide strong interaction with the hosting cured epoxy matrix, and the “core” part similar to the structure of CFs, and hence able to establish strong π-π interactions with the reinforcing CFs. The article also covers the effect of functionalized graphene oxide incorporation on the mechanical, thermal, electrical, and viscoelastic properties of composite materials reinforced with carbon fibers.

## 1. Introduction

Owing to their low specific weight, high stiffness, superior temperature and corrosion resistance, and ease of processability, carbon fiber-reinforced polymeric (CFRP) composites are used in broad range of applications from aerospace to marine industries [1]. CFRPs display excellent tensile strength; however, the brittleness, crack-prone matrix and the fiber–matrix interface with a weak adhesion usually lead to a failure of the composite structures in terms of delamination and catastrophic failure [2,3]. In this direction, carbon nanostructured forms (CNFs) can help to overcome these criticalities. The use of CNFs in bulk epoxy resins and their composites has been pursued by many authors, not only to increase the mechanical performance of the resulting composites but also to confer them functional properties, such as de-icing, self-sensing, and self-healing, or to save energy during the manufacturing process [4,5,6,7]. Multi-wall carbon nanotubes (MWCNs) have been successfully incorporated in CFRCs, imparting them electrical conductivity, whose value depends on the process adopted for the manufacturing [8,9]. Graphene-based nanoparticles are expected to further improve, with respect to CNTs, some functional properties depending on the thermal conductivity of the composite (thermal management), and to reduce the humidity content in composites based on epoxy resins [10]. For these reasons, many authors have made tremendous efforts to obtain graphene in the form of single layers, for example, training to completely exfoliate graphite particles (to reach almost 100% of exfoliation). Despite the large efforts made to obtain and employ perfect and almost single graphene layers, no high-performance materials, in terms of electrical properties and related functional properties, have been obtained. This is because defect-free single graphene layers tend to reassemble during the steps of nanocomposite manufacturing. To avoid reassembling arrangements, functionalization procedures are needed. This allows the attaching of chemical groups to the graphene layers able to prevent the re-assembling because of steric and energy factors. If the functionalization is performed through functional groups covalently bonded to the single graphene layers, the reassembling phenomena are prevented, but the change in the hybridization state of the carbon atoms, from sp^2^ to sp^3^, in single layers results in the partial loss of the phenomenon of electron delocalization of carbon nanoparticles and, as a consequence, in the impoverishment of their electronic properties [11]. To meet this scientific and technical challenge, it is possible to use graphene oxide nanosheets, which are constituted of the stacking of different graphene layers (able to manifest the desired phenomenon of electron delocalization) and polar functional groups on the edges of these thin graphene-based blocks. GO nanoparticles dispersed in the polymeric matrix are expected to also improve the interfacial adhesion between woven carbon fibers and the epoxy matrix during the impregnation process of the woven fibers. In fact, GO nanoparticles are composed of polar groups, especially on the edges of the nanosheets, able to provide strong interaction with the hosting cured epoxy matrix (composed of a large number –OH polar groups) and the “core” part, similar to the structure of CFs, therefore being able to establish attractive interactions with the reinforcing CFs of the woven fibers. This is a hot topic worth investigating for the obvious applicative implications. There are many relevant papers in the literature that have revealed that the presence of GO nanosheets in the epoxy resin should allow the conferring of functional properties to the resin, whereas, in other papers, GO should promote better adhesion between carbon fibers and the polymeric matrix. This review aims to investigate recent advances achieved in this last direction. To improve the interfacial properties of the composites, a resilient interfacial adhesion between the fiber and matrix plays a significant role. In recent years, a series of methods have been introduced to improve the interfacial adhesion between the fiber and matrix such as the coating of fibers, sizing, 3D weaving, and tufting [12]. Unfortunately, these approaches are only applicable to textile laminates made with resin transfer molding (RTM) procedures, and are thus not applicable on prepreg laminates [13,14]. The poor defective surface of carbon fiber lacks the functional groups on its surface and thus has insufficient wettability and interaction with the polymer matrix. Functional groups such as hydroxyl –OH, carboxyl –COOH, and carbonyl –CO are added on the surface of CFs or in the epoxy matrix to boost their functionality as a CFRP reinforcement [15]. The effect of the functionalization of carbon fibers or epoxy with nanoparticles such as carbon nanotubes, nano clay, and graphene nanoplatelets has been investigated in detail [16,17,18,19,20,21,22,23,24,25,26]. However, control of the carbon nanotubes (CNTs) and nano clay dispersion is difficult and not cost-effective. In addition, their agglomeration onto a fiber surface leads to reductions in the glass transition temperature (T_g_) and mechanical properties of the developed composites [27]. Recently, to attain good interfacial adhesion, the modified graphene was significantly incorporated in CFRP composites [28]. The improvement in the thermal conductivity of the CFRP by grafting carbon fibers with a 3D graphene network was examined, where an increase in thermal conductivity of about 165% in comparison with that of pure CFRP was observed for the graphene-incorporated composites [29]. The fatigue behavior and mode I fracture toughness of CFRP functionalized by graphene nanoplatelets (GNPs) was investigated, and it was observed that with 0.1% of GNPs, the mean fatigue life and mode I interlaminar fracture toughness increased to 155% and 40%, respectively [30]. A strong interaction was observed in the case of GO-incorporated composites as compared to unfunctionalized graphene sheets-incorporated composites due to the abundance of various functional groups [31]. The use of graphene oxide functionalization with polymers has gained momentum after this development. The functionalization of graphene oxide resulted in an improvement in interfacial adhesion in terms of preventing delamination and crack propagation. This review article emphasizes graphene-promoted (with better interfacial adhesion) carbon fiber-reinforced epoxy composites and differentiates it from graphene/polymer composites or graphene-based fibers in polymer composites. Herein, we discuss different routes of functionalization of GO nanosheets and their incorporation onto fiber surfaces or in polymer matrixes, helping to improve the interfacial adhesion between the fiber and matrix, and thus helping to improve the mechanical, thermal, electrical, and viscoelastic properties of CFRP-based composite materials.

## 2. Functionalization of Graphene Oxide Nanosheets

**Figure 1:**
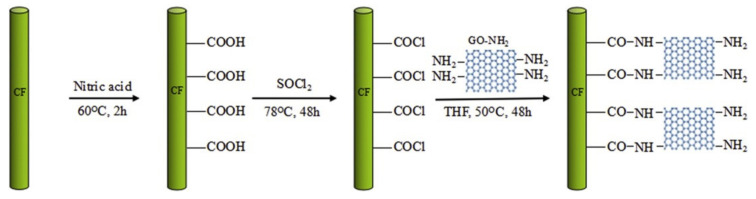
Schematic procedure of CF treatment [38]. Reproduced with permission from Materials & Design; published by Elsevier, 2016.

Intermolecular attractive forces at the interface generate an intrinsic weak zone at the nanoscale, which leads to delamination in CFRPs. To improve the molecular interaction between the fiber and matrix at the CFRP interface, the surface energy can be changed by either (1) increasing the bonding sites on the surface or (2) activating the surface energy using chemicals [32,33]. To increase load transfer across the fiber/matrix interface, it is critical to establish extensive dispersion of the GO, as well as strong interfacial contacts between the fiber and matrix, while inserting individual nanomaterials [34,35]. GO has a variety of functional groups, including hydroxyl, carboxyl, carbonyl, amine, and epoxide groups, which serve as covalent functionalization reaction sites. Furthermore, because these functional groups have a strong affinity for polar aprotic solvents, GO is easily distributed and, as a result, GO could be employed in bonded joint composites. The GO surface has potential covalent functionalization, which leads to an increase in the interfacial contact between the GO and the polymer matrix [36,37]. Several researchers are focusing on determining the effect of functional groups on the interfacial properties of polymer composites. Polymer composites via direct grafting of the carbon fiber surface with GO-NH_2_ (amino-functionalized graphene oxide) were fabricated, where the authors focused their investigation onto the interfacial shear strength and tensile properties of the composite laminates. The grafting procedure first includes the oxidation of carbon fiber surfaces with nitric acid to obtain acid groups, and then fibers are treated with thionyl chloride to obtain acyl chloride-functionalized carbon fibers (CF-COCl), which are later treated with GO-NH_2_ to achieve a carbon fiber surface decorated with GO-NH_2_ (Figure 1) [38].

**Figure 2:**
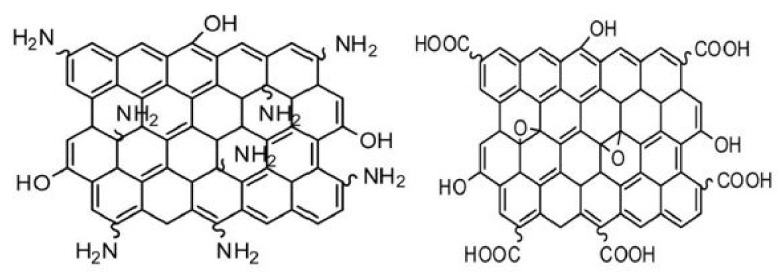
GO sheet functionalized with amine, carboxyl, and hydroxyl groups [41]. Reproduced with permission from International Journal of Biological Macromolecules; published by Elsevier, 2018.

Chen and co-workers studied the effect of silanized GO on the interface of an epoxy/carbon fiber where laminates were prepared with a constant volume percent of unidirectional carbon fibers cured in epoxy through RTM. The data showed that laminates prepared with unfunctionalized GO nanoparticles (0.5 wt.%) showed decreases in interfacial shear strength (IFSS), interlaminar shear strength (ILSS), and flexural and tensile strength in comparison with the base composite without nanoparticles, whereas the laminates prepared with silanized GO showed increases in the IFSS, ILSS, flexural strength, and flexural modulus by 60%, 19%, 15%, and 16%, respectively, due to effective stress transfer, energy absorption, and controlled crack propagation provided by silanized GO in the interface [39]. In another study, epoxy nanocomposites by incorporating silanized graphene oxide in a 3-aminopropyltriethoxysilane (APTS) epoxy matrix were prepared. The GO was silanized by using organosilane and covalently functionalized with epoxy; it was well known that silanized GO contains hydroxyl and carboxyl groups on their basal planes and edges, as shown in Figure 2. The active sites reacted with APTS amine groups and resulted in strong interfacial adhesion of the nanocomposites. The tensile strength and elongation in failure of nanocomposites were increased to 45% and 133%, respectively. The thermal stability of epoxy resin containing silanized GO was also found higher in comparison with untreated epoxy resin [40]. Figure 2 illustrates different functional groups-decorated graphene nanosheets. The presence of NH_2_ terminal groups was able to promote crosslinking reactions with the epoxy precursors, making the filler chemically anchored with the polymer matrix. 

## 3. Functionalization of (Fiber/Polymer/Symbiosis) Using Different Groups-Decorated GO Nanosheets

**Figure 3:**
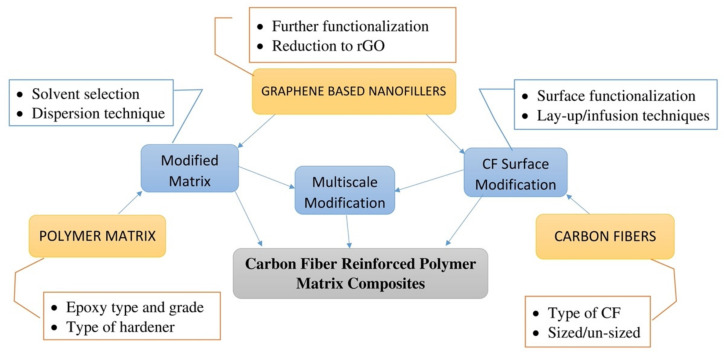
Flowchart for manufacturing GO-functionalized CFRPs.

The improvement in the mechanical performance of the composite laminates was achieved by increasing the interfacial adhesion between the fiber and the epoxy matrix. To alter the properties of CFRP, either the modification of the fiber surface or epoxy or even both can be carried out [42]. Figure 3 depicts the basic flowchart for the manufacturing of graphene oxide-modified CFRPs, including the modification types and points to be considered during functionalization.

### 3.1. Modification of Carbon Fibers Using Different Groups-Decorated GO Nanosheets

**Figure 4:**
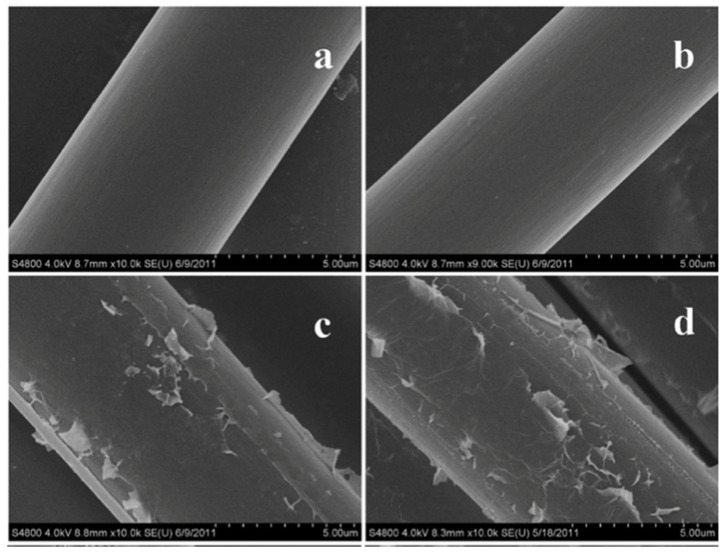
SEM images of (**a**) pristine carbon fiber; (**b**) CF-GO0; (**c**) CF-GO1; (**d**) CF-GO5 [45]. Obtained with permission from American Chemical Society; published by ACS Publications, 2012.

Functionalized carbon fiber-reinforced polymer composites demonstrate enhanced mechanical and thermal properties [43,44]. The argument was supported by Zhang et al. by decorating carbon fibers with GO sheets, investigating the mechanical performance of GO-reinforced CFRP composites, and finding a significant alteration in IFSS, ILSS, and tensile properties of the composite panel. The dispersion of GO was also achieved and verified through scanning electron microscopy (SEM) images, as shown in Figure 4. The concentration of 5 wt.% resulted in the agglomeration of GO particles on the surface of the carbon fibers [45].

The researchers also analyzed some grafting methods of GO on carbon fiber surfaces such as the continuous coating process, liquid phase deposition strategy, and sizing agent application. The advantage achieved through these processes were a uniform dispersion of GO, increased electrical conductivity, and shielding against interfacial oxidation [46,47,48]. In another study, the coating of GO on a CF surface was also reported by applying the electrophoretic deposition method for improving the electromagnetic interference (EMI) shielding. In this process, graphene-charged particles were deposited on the surface of conductive carbon fibers under an electric field and resulted in the formation of a thin layer over the surface. The incorporation of this coated CF in the epoxy matrix resulted in a 16.3% increase in EMI effectiveness [49,50].

**Figure 5:**
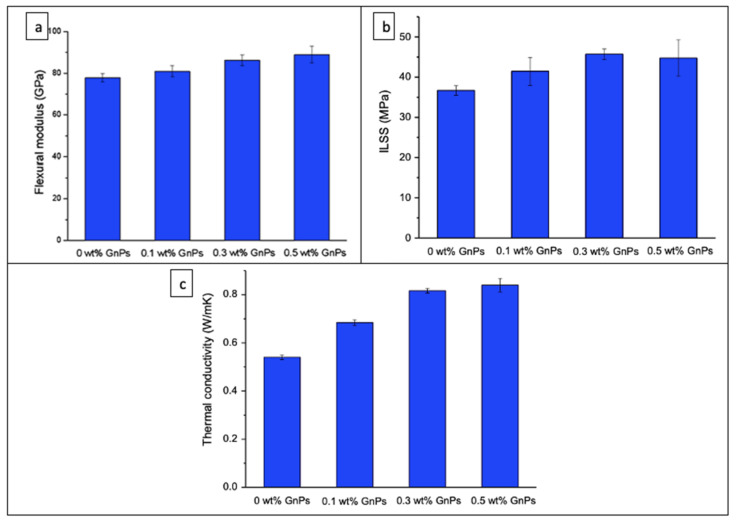
(**a**) Flexural strength; (**b**) interlaminar shear strength; (**c**) thermal conductivity variation at different wt.% of GNPs [55]. Obtained with permission from Journal of Materials Science; published by Springer Nature, 2018.

Multi-functional composites can be prepared by utilizing the synergetic effect of different ingredients used for functionalization. Researchers encountered the effect of different ingredients such as polydopamine (PDA), branched polyethyleneimine, and diamines (D_400_)-mixed graphene oxide functionalization on the CF surface and found a significant improvement in tensile strength, ILSS, and mode II fracture toughness of the composites in comparison with pristine CF and nonfunctionalized CF/epoxy composites [51,52,53]. A composite laminate with a cyanate ester resin reinforced with unidirectional carbon fibers was fabricated and functionalized with graphene nanoplatelets by using solution blend and pre-soak methods. The results showed an abrupt increment in thermal and electrical conductivity of the composite with GNPs incorporation [54]. The effect of GNPs-coated carbon fiber incorporation onto epoxy composites was also examined, and an increment in flexural strength, interlaminar shear strength, and through-plane thermal conductivity with 0.3 wt.% of GNPs incorporation was observed (Figure 5) [55].

The enhancement in properties was achieved by improved interfacial properties of the composites. To attain excellent interfacial properties, a composite laminate with modified carbon fibers by depositing GO/SiO_2_ particles layer-by-layer was prepared and observed to increase the IFSS, ILSS, and flexural strength by 86.1%, 89.3%, and 30.4%, respectively, compared with the untreated carbon fiber composite [56]. A solvent treatment method was studied by treating GO with divinylbenzene (solvent) and infusing the suspension on the carbon fiber surface. The method resulted in an enhanced ILSS and electrical conductivity to about 76% and 150%, respectively [57].

As a result, it can be stated that fiber modification via graphene oxide functionalization improved the interaction energy between carbon fibers and polymer adhesives. Furthermore, the functional groups on the surface of carbon fibers, such as hydroxyl and carboxyl groups, reacted with epoxy groups on GO sheets, assisting in the formation of a chemical bond between carbon fibers and GO sheets; thus, a combination of these factors aids in the improving interfacial adhesion between the fiber/matrix interphase.

### 3.2. Modification of Polymer Matrix Using Different Groups Decorated GO Nanosheets

**Figure 6:**
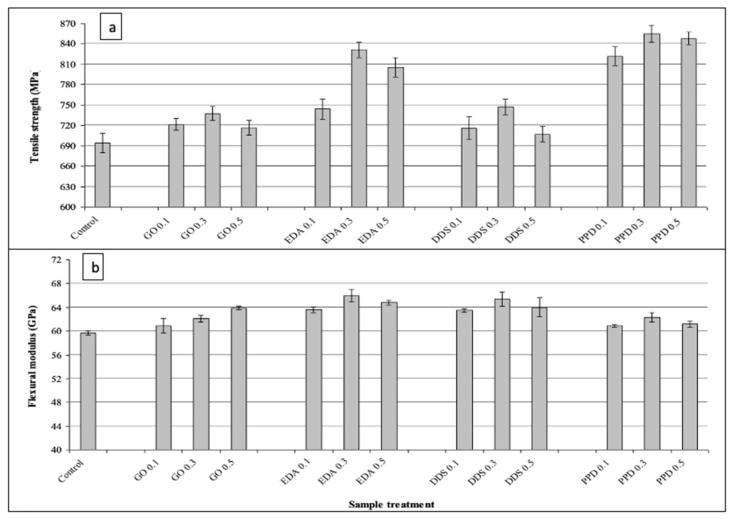
(**a**) Tensile strength; (**b**) flexural strength for different-solvents-treated GO/epoxy/CF composites [60]. Obtained with permission from Polymer Testing; published by Elsevier, 2015.

Epoxy resins are widely utilized in the manufacture of lightweight CRFPs to provide desired technical features such as high modulus and strength, low creep, and excellent chemical and thermal stability [58]. Matrix alteration can be used to improve mechanical and/or other qualities and the key for that is a uniform dispersion of GO nanosheets in an epoxy resin [59]. An epoxy resin was prepared with GO functionalized by different molecules such as ethylenediamine (EDA), 4,4′-diamino diphenyl sulfone (DDS), and p-phenylenediamine (PPD) and cured with carbon fibers. The results demonstrated an improvement in tensile and flexural properties when compared with nonfunctionalized GO-CFRP composites (Figure 6) [60].

**Figure 7:**
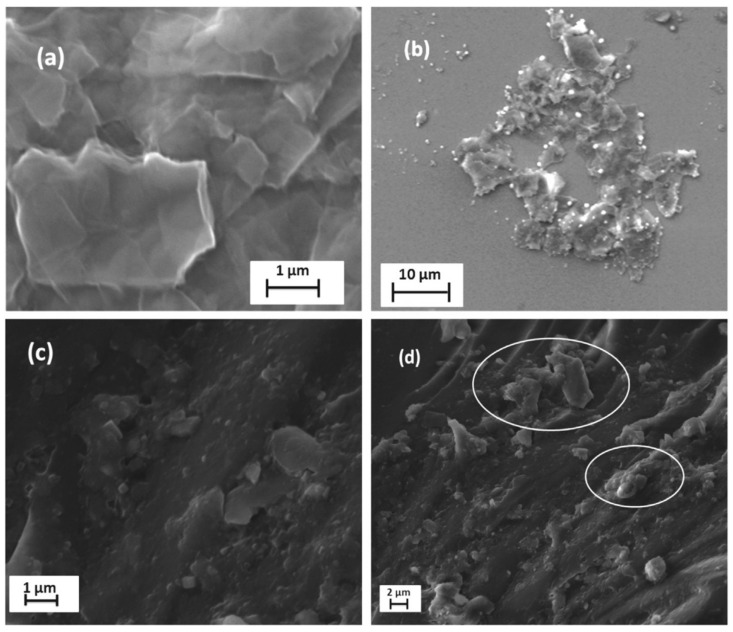
SEM images: (**a**) GO flakes; (**b**) dispersed GO flakes; (**c**) GO flakes dispersed in epoxy; (**d**) agglomeration of GO flakes in resin [67]. Obtained with permission from Composites Science and Technology; published by Elsevier, 2016.

Later on, the effects of polyethyleneimine-treated and hydrazine-reduced GO methods were also examined, and the result showed that the incorporation of 0.2–0.3 wt.%-functionalized GO in the epoxy resin resulted in excellent in-plane and out-of-plane properties [61,62]. Polyurethane foams by incorporating an isocyanate matrix functionalized with graphene oxide were manufactured. In this method, the final nanocomposite foam was divided into two components. Component A represented the matrix solution of polyether polyol and polyester polyol and component B represented the mixture of polyaryl polymethylene isocyanate (PAPI) with graphene oxide particles. The method resulted in an improvement in thermal conductivity of the order of 0.87 W/mK [63]. On the other hand, the development of some dispersion techniques was also carried out to achieve a uniform dispersion of GO throughout the matrix material. The GO-functionalized epoxy/CF composites were prepared by homogenous dispersion through the sonication method and were followed by the resin infusion technique. The method resulted in an appropriate dispersion of GO with a 0.5 wt.% content and achieved excellent tensile and flexural strengths of 519 MPa and 525 MPa, respectively [64]. The CFRP composite laminate by incorporating carbon fibers with functionalized (GO) epoxy resin was also prepared through the ultrasonication technique. In this technique, first, the GO flakes were mixed with ethanol and ultra-sonicated for 5 h, and then, ethanol was evaporated by keeping the GO/epoxy mixture in a vacuum oven for 5 h at 60 °C. The technique resulted in improved interfacial properties of the composite laminates [65]. A CFRP laminate by incorporating unidirectional carbon fibers in an epoxy/curing agent mixture along with functionalized GO through a high-speed planetary mixing method was also examined. In this method, the GO/epoxy was mixed by using a dual asymmetric centrifugal speed mixer, resulting in a uniform dispersion of GO due to the shear forces generated within the mixture. The increased electrical and thermal conductivity supported the method being robust for achieving a good dispersion [66]. Similarly, the dispersion of GO and its effect on flexural and interlaminar shear strengths at different weight percent concentrations were investigated (Figure 7). It was found that with 0.3 wt.% of GO, the flexural strength and ILSS increased by 66% and 25%, respectively [67].

To attain the multi-functional properties of CFRP composites with a uniform dispersion of nanoparticles, the three-roll milling (TRM) method can also be applied. In this method, a mixer of epoxy and nanoparticles without hardener was passed through three rollers with different rotational speeds such as N_1_ for the feed roller, N_2_ for the central roller, and N_3_ for the apron roller, and exfoliated a uniform dispersed GO. This method was found far better than the shear mixing and ultra-sonication method in terms of higher out-of-plane properties without additional solvents [68].

This section demonstrated the effect of matrix modification with functionalized graphene oxide and the methods used for attaining a uniform dispersion of GO throughout the matrix. It has been illustrated that the addition of GO up to 0.3 wt.% was the most optimum amount for improving the interfacial adhesion of the composite materials due to better load transfer from the matrix to the CFs. Furthermore, GO addition results in the agglomeration of nanoparticles and decreased bonding strength.

### 3.3. Modification of Both (Fiber/Matrix) Using Different Groups-Decorated GO Nanosheets

The multi-scale modification of carbon fiber/epoxy composites with graphene-based nanomaterials is the most intriguing field of current studies. The addition of nano-fillers to the fiber surface and resin system can simultaneously have a direct impact on the composites mechanical, thermal, and chemical properties. As a result, the material’s strength, dimensional properties, and functional life are extended [69,70]. CFRP composite laminates by functionalizing graphene oxide were prepared through two routes: (i) Mixing the GO directly with the epoxy via the TRM method. GO was mixed with 0.5 and 1 wt.%, and then the solution was mixed with hardener and degassed at room temperature for 1 h in vacuum. (ii) Spraying the carbon fiber surface with ethanol-mixed GO, obtaining a 0.5 and 1 wt.% deposition of GO, and then drying the fibers at 70 °C for 30 min to remove the solvents. The above-mentioned process resulted in a good interlaminar shear strength of the laminate; however, certain parameters need to be controlled during processing such as the controlled spraying of GO, epoxies viscosity, and functional group presented on the GO surface [71].

**Figure 8:**
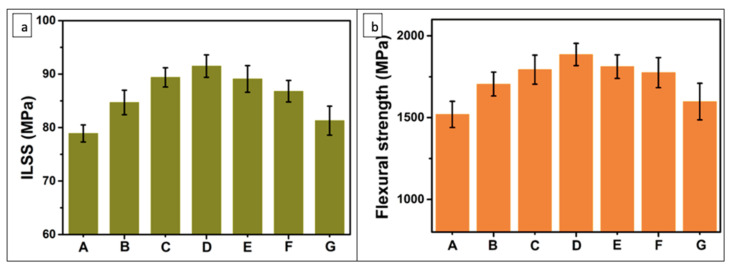
(**a**) Interlaminar shear strength; (**b**) flexural strength of composites: (A) untreated CF, (B) CF/GO (0.1%), (C) CF/GO (0.3%), (D) CF/GO (0.5%), (E) CF/GO (0.8%), (F) CF/GO (1.0%), (G) CF/GO (0.5% one step) GO wt.% content [73].

Researchers also encountered the rheological properties of different carbon-based functional elements such as GO, reduced graphene oxide (rGO), GNPs, and multi-wall carbon nanotubes (MWCNTs) reinforcement with epoxy and fiber simultaneously. The data showed that the viscosity behavior of pure epoxy was Newtonian for the suspension, and it became non-Newtonian for MWCNTs-reinforced CFRP composites, whereas, for GO, rGO, and GNPs, it still behaved Newtonian [72]. In another approach, carbon fibers were treated with an aqueous solution of phthalazinone ether sulphone mixed with GO flakes, and the simultaneous deposition of GO layers (0, 0.1, 0.3, 0.5, 0.8, 1.0, 0.5 one-step) on CF was carried out. The results showed that with a 0.5 wt.% content of GO, the ILSS and flexural strength of treated CF/epoxy matrix was 92 and 1886 MPa, respectively; thus, it was higher than that of untreated CF/epoxy laminates (Figure 8). However, the GO content beyond 0.5 wt.% tended to decrease the properties slightly due to agglomeration in the interfacial region. By contrast, the advantage of the experiment was evaluated by depositing GO aqueous solution directly on CF and resulted in a lower ILSS (81.3 MPa) and flexural strength (1525 MPa) in comparison with the 0.5 wt.% CF/GO composite [73].

Recently, in aerospace industries, CFRP composites have been manufactured using the hand lay-up technique followed by autoclave curing by the multiscale functionalization of the CF and epoxy matrix. In this process, first, the carbon fiber prepregs were treated with graphene-based nanoparticles and then cured with epoxy resin. The mixture of GO/epoxy resin was achieved by using the shaft-mixing method operating at 2000 rpm for 60 min, followed by a Silverson high-shear batch mixer at 3000 rpm for 30 min. The above-mentioned process resulted in the homogeneous distribution of graphene nanoparticles in the epoxy resin, thus helping to achieve better shear and flexural strength [74].

The multi-scale modification of fiber and polymer helps to improve the interfacial adhesion by combining the effect of functionalization on the fiber surface, as well as in the polymer matrix. However, certain parameters (viscosity of epoxy, functional groups on GO surface, controlled spray, etc.) need to be monitored for a uniform deposition and dispersion of GO nanoparticles. Therefore, additional research needs to be carried out in the area for improving and analyzing the mechanical and rheological performances of the GO-promoted adhesion properties, required for achieving the deigned CFRP composites.

## 4. Effect of Functionalization on CFRPs Properties

The effect of functionalized graphene nanosheets incorporation on CFRPs has been summarized in terms of their mechanical, rheological, and conductive properties [75].

### 4.1. Mechanical Properties

**Figure 9:**
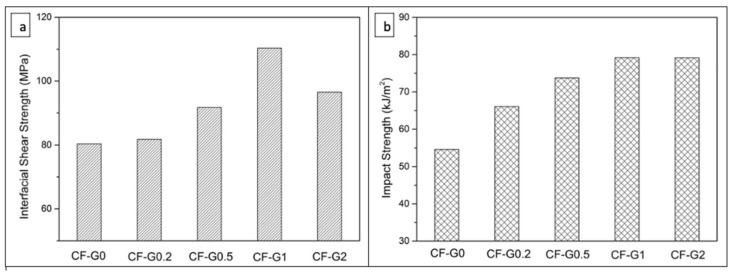
(**a**) Interfacial shear strength; (**b**) impact strength variation with (0, 0.2, 0.5, 1, 2) GO wt.% content [79]. Obtained with permission from Applied Surface Science; published by Elsevier, 2018.

The addition of graphene nanosheets in CFRP composites results in a significant improvement of mechanical properties such as tensile strength, interfacial shear strength, interlaminar shear strength, and fracture toughness [76,77]. It was observed that the GO/CF-ECO/epoxy composite showed a 50% improvement in IFSS than virgin GO/CF/epoxy did [78]. The CFRP composite with varying GO content (0 to 2 wt.%) was prepared and examined for IFSS and impact strength of the various samples. Figure 9 shows the variation in IFSS and impact strength with different GO (0, 0.2, 0.5, 1, 2 wt.%) contents [79]. Table 1 summarizes the mechanical properties for the functionalized GO nanosheets-incorporated CFRPs.

### 4.2. Thermal Conductivity

**Figure 10:**
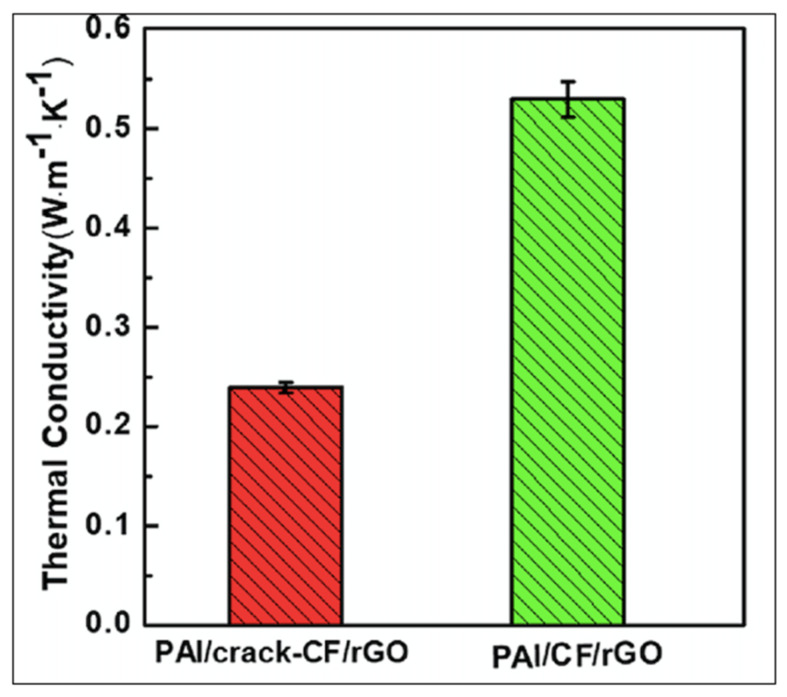
Thermal conductivity of cracked and uncracked carbon fiber-reinforced composites [29]. Obtained with permission from Composites Part B: Engineering; published by Elsevier, 2019.

The addition of functionalized GO nanosheets in composite materials results in the enhancement in thermal conductivity [80], where the thermal diffusivity and specific heat of the materials are the key parameters for measuring the thermal conductivity (Equation (1)).

(1) $$k=\rho \alpha C_{p}$$

where *k* is the thermal conductivity (W/mK), *ρ* is the material density (kg/m^3^), *C_p_* is the specific heat (J/kg K), and *α* is the thermal diffusivity across the thickness (m^2^/s). The thermal conductivity of functionalized epoxy resin incorporated with carbon fibers was examined. A study found that the addition of 1.0 wt.% graphene nanoparticles is useful to improve the thermal conductivity from 0.68 to 0.72 W/mK [16]. Polyamide-imide reinforced with cracked and uncracked carbon fibers along with functionalized reduced graphene oxide composites were prepared, and its effect on thermal conductivity was examined. It was found that an uncracked network of graphene particles creates a more efficient thermally conductive path in comparison with cracked fibers. The effect of fiber dimensions (chopped and continuous) on the thermal conductivity is shown in Figure 10 [29]. The effect of functionalization on the thermal conductivity of the CFRP laminates is listed in Table 2.

### 4.3. Electrical Conductivity

**Figure 11:**
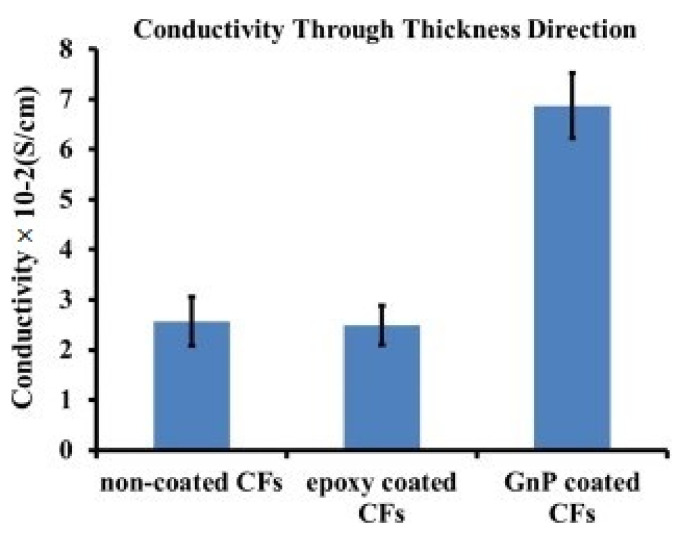
Electrical conductivity of noncoated, epoxy-coated, and GNPs-coated carbon fiber surfaces [46]. Obtained with permission from Composites Part B: Engineering; published by Elsevier, 2015.

Regarding electrical conductivity, a poor electrical conductivity is observed for nanocarbon fiber composites. The addition of GO nanosheets in CFRP tends to improve its electrical conductivity, where the through-the-thickness resistivity can be calculated by using Equation (2).

(2) $$\rho =\frac{RAc}{h}$$

where *ρ* is the resistivity (Ωm), *R* is the resistance (Ω), *Ac* is the effective area of the electrode, and *h* is the thickness of the samples. In a study, 3 mm-thick samples were tested for electrical conductivity, which showed that the addition of 1.0 wt.% graphene content enhanced the electrical conductivity from 4.3 × 10^−15^ to 2.6 × 10^−6^ S/m [16]. A CF/epoxy composite by coating the carbon fiber surface with GNPs was manufactured and compared with noncoated CFs and epoxy-coated CFs. The layer thickness for the epoxy and GNPs coating was 600 nm, and it resulted in the higher conductive path through the thickness of carbon fibers, as shown in Figure 11 [46]. Table 3 represents the effect of functionalized GO nanosheets incorporation on the electrical conductivity of CFRPs.

### 4.4. Viscoelastic Properties

**Figure 12:**
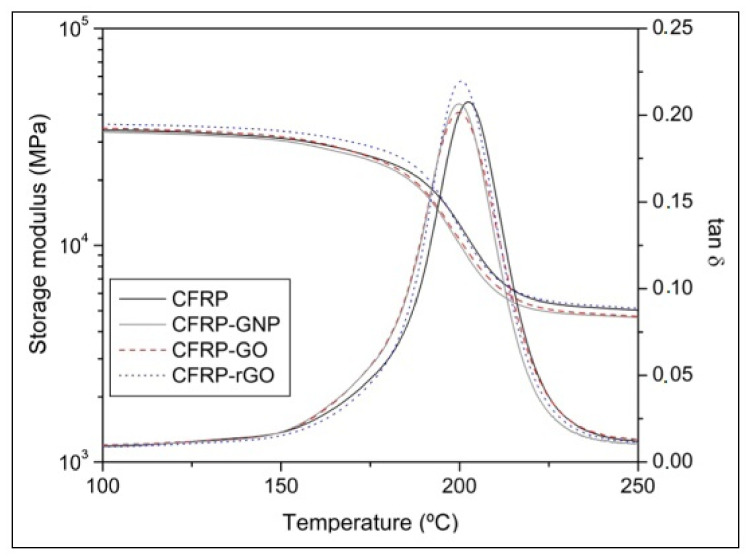
Storage modulus and tanδ curve for different nanoparticles [78].

When dealing with the mechanical properties of polymeric materials, one of the most important parameters to examine is their viscoelastic behavior. This characteristic determines polymer elasticity when stress is applied, resulting in composite recovery. The viscoelasticity property is the reorganization of molecules in the polymer composite after the stress–strain cycle [81]. The effect of different graphene-based nanoparticles on the glass transition temperature of the laminated composites was determined. Figure 12 covers the addition of GO and rGO, and the interaction of oxygenated groups with an epoxy precursor leads to the creation of a covalent bond between the resin and nanoparticles, and thus a nonstoichiometric balance occurs in the resin. This tends to slightly decrease the glass transition temperature with respect to the CFRP sample, as expected for samples where a small fraction of epoxy resin is cured under nonstoichiometric conditions (defect of hardener) [10]. Table 4 summarizes the viscoelastic behavior and glass transition temperature (T_g_) of various CFRP composites [78].

## 5. Applications of Carbon Fiber-Reinforced Polymer Composites

**Figure 13:**
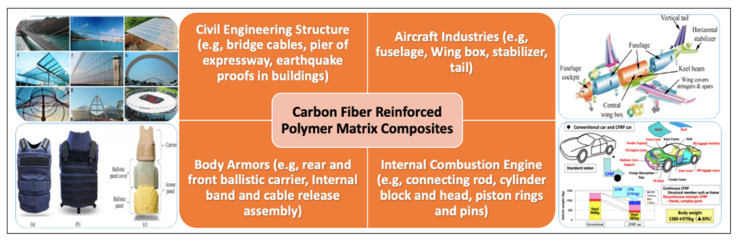
Applications of carbon fiber-reinforced polymer composites [86,87,88,89].

From aerospace to automotive industries, carbon fiber-based polymer composites can contribute a vital role as these applications necessitate a lightweight material with outstanding mechanical performance, high stiffness, corrosion resistance, low coefficient of thermal expansion, chemical resistivity, and excellent electrical and thermal conductivity. Temperature changes and adverse atmospheric conditions, such as saline water and high wind, must be tolerated by the developed composite materials. Multifunctional composites might be quite intriguing in this scenario; thus, combining the excellent mechanical qualities of CF/epoxy composites with other embedded functionalities such as temperature management [4], energy storage, and sensing properties [82] would result in a technical breakthrough in the applicability of carbon-based composites [83]. In the design of airplanes and spacecrafts, the risk of destruction due to impact was well understood. To negotiate these effects, the introduction of CFRP composites is increasing rapidly; for example, an A-320 aircraft has a 21.5% composite usage to its total weight, and a Boeing 787 and Airbus A350 have 50% of its total weight comprised of CFRP for different parts including the tail cone, center wing box, vertical and horizontal tails, and pressure bulkheads. On the other hand, the usage of CFRP composites was also started in the field of military aircraft due to their outstanding performance and the excellent strength-to-weight ratio [84,85]. Figure 13 depicts the sectors for the application of CFRP composites.

The use of CFRP composite laminates has also been initiated in the area of civil engineering and structural parts such as stock cables for bridges and suspension bridges. The cables are used for replacing the conventional steel cables throughout the world due to their nonrelaxing, noncorroding, light-weight quality, and stress-free behavior. Table 5 represents the comparison of properties of CFRP cables in comparison with that of conventional steel cables. It has been found that the CFRP cable shows excellent mechanical performance in terms of elastic modulus, tensile strength, and density [89].

The automotive sector also started using CFRP composites due to their anisotropic distribution of mechanical properties, advantageous for the fabrication of certain engine components such as conrods R4 (82/71). The material is made up of two monolayer polymers with fibers, oriented differently. The conrod’s main body and cap are constructed of two monolayer polymers that are combined in various quantities [90]. In today’s environment, there is a growing demand for lightweight, flexible, and durable body armor that can provide enhanced ballistic protection, particularly against the increasingly lethal threats that soldiers will face. CNTs and graphene, which are among the world’s strongest and stiffest materials, will certainly contribute in the development of ultra-strong, massive-energy-absorber, lightweight, and robust composites for future body armor construction. High-performance carbon fiber-reinforced polymers functionalized with graphene-based nano-fillers are currently used in ballistic systems as hybrid nanocomposites to construct the ultimate body armor of the future [91].

## 6. Conclusions

The use of functionalized GO nanosheets aims to improve the interfacial adhesion among fibers and matrix, thus helping to improve the overall properties of CFRP composites including mechanical, thermal, electrical, and viscoelastic properties.

Herein, the effect of functional groups involvement in the covalent functionalization of CFRP has been covered, and it was observed that GO open reaction sites to the fiber surface and epoxy matrix. The terminal hydroxyl groups provide a globular dendritic and void-containing topology to the fiber surface, and the carboxyl groups oxidize the fiber surface; both provide many open spaces and allow the carbon fibers to interlock perfectly between the resins. Among different functionalization approaches, GO functionalization with silane coupling agents provides an excellent route as it produces carboxyl, hydroxyl, and amine groups on the surfaces and edges and results in improved interfacial adhesion of the composites. 

The proper dispersion of graphene oxide in the polymer matrix can be carried out via three-roll milling (TRM), shear mixing, and ultra-sonication where TRM attains a more uniform dispersion in comparison with shear mixing and ultra-sonication due to their ease of processing without any additional solvent. However, a uniform dispersion of GO nanosheets in a matrix or fiber surface is still a challenge, and in the case of fiber surface modification, compatibility is high, resulting in better composite interfacial adhesion compared to matrix/GO modification.

The review also covers the applications of these modified carbon fiber-reinforced polymer composites in aircraft, automobile, body armor, and civil engineering sectors with improved overall composites performance using GO-promoted interfacial adhesion.

## Figures and Tables

**Table 1:** Listed mechanical properties of CFRPs with functionalization.

Composite	Fiber	Matrix	Modification Type	Processing Technique	Mechanical Properties (without Functionalization)	Mechanical Properties (with Functionalization)	References
CF/GO/epoxy	AS4 3K 8HS carbon fiber	1.0 wt.% GO/Epon 828 epoxy resin and Epicure W curing agent	Matrix modified	Compression molding	Mode I fracture toughness (GIC)-191.4 J/m^2^ Mode II fracture toughness (GIIC)-978.9 J/m^2^	Mode I fracture toughness (GIC)-203.2 J/m^2^ Mode II fracture toughness (GIIC)-917.9 J/m^2^	[16]
CF/GO/epoxy	T300 carbon fiber	0.3 wt.% GO/epoxy resin LY556 and triethylenetetramine (TETA) hardener	Matrix modified	Hydraulic pressing	Flexural strength-425 MPa ILSS-32.2 MPa	Flexural strength-710 MPa ILSS-40.8 MPa	[67]
CF/GO/PI	IM7 8552 carbon fiber	Laser-induced GO array/Polyimide substrate	Matrix modified	Manual pressured roller	Short beam strength-121 MPa Mode I fracture toughness (GIC)-292 J/m^2^ Mode II fracture toughness (GIIC)-1.3 KJ/m^2^ Tensile strength-2.0 GPa	Short beam strength-122 MPa Mode I fracture toughness (GIC)-425 J/m^2^ Mode II fracture toughness (GIIC)-1.5 KJ/m^2^ Tensile strength-2.5 GPa	[12]
PAI/CFs/GO	GO/PAN-based carbon fiber	Polyamide-imide	Fiber modified	Compression molding	Tensile strength-34.13 MPa Tensile modulus-3.03 GPa Fracture toughness-101.11 J/m^3^	Tensile strength-38.41 MPa Tensile modulus-2.22 GPa Fracture toughness-106.40 J/m^3^	[29]
SCF-GO7.5/epoxy	5.0 wt.% GO/T700S carbon fiber	E20 and E50 epoxy resin	Fiber modified	Autoclave	Interfacial shear strength-72.0 MPa Interlaminar shear strength-45.5 MPa Tensile strength-1446.7 MPa	Interfacial shear strength-97.2 MPa Interlaminar shear strength-51.3 MPa Tensile strength-1942.1 MPa	[45]
GO/CF/epoxy	0.5 wt.% GO/carbon fiber	MGS RIM 135 epoxy resin and Rim 37, RIM134 hardener	Fiber modified	Vacuum-assisted resin infusion molding	Tensile strength-1525 MPa Young’s modulus-5.2 GPa ILSS-65.4 MPa	Tensile strength-1726 MPa Young’s modulus-8.6 GPa ILSS-52.6 MPa	[63]
ACF- D400-GO	GO/T700SC carbon fiber	D400	Fiber modified	Hydraulic pressing	Tensile strength-6.24 GPa Interfacial shear strength-91.3 MPa	Tensile strength-5.02 GPa Interfacial shear strength-82.2 MPa	[39]
rGO/CF-U	GO/T700SC carbon fiber	-	Fiber modified	Electrophoretic deposition	Tensile strength-5.06 GPa Interfacial shear strength-53 MPa	Tensile strength-6.11 GPa Interfacial shear strength-79.5 MPa	[50]
LBL 10 Go/CF/PP	10 layer GO/Carbon fiber	Polypropylene	Fiber modified	Knotting-melting	Tensile strength-5.2 GPa Interfacial shear strength-4.4 MPa	Tensile strength-5.3 GPa Interfacial shear strength-10.1 MPa	[52]
PDA-GO-CF/epoxy	GO/T300 carbon fiber	Epolam 5015 epoxy resin and amine hardener	Fiber modified	Wet lay-up	Mode I fracture toughness (GIC)-630 J/m^2^ Mode II fracture toughness (GIIC)-1625 J/m^2^	Mode I fracture toughness (GIC)-900 J/m^2^ Mode II fracture toughness (GIIC)-1720 J/m^2^	[51]
rGO/CF/epoxy	0.2 wt.% GO/Plain weave carbon cloth	LAPOX C51 epoxy resin and lapox AH-428 hardener	Fiber modified	Vacuum assisted resin transfer molding	Interlaminar shear strength-23.3 MPa In-plane fracture toughness (kIC)-20.6 MPa.m1/2 Impact toughness-5.2 J	Interlaminar shear strength-43 MPa In-plane fracture toughness (kIC)-27.3 MPa.m1/2 Impact toughness-10.4 J	[62]

**Table 2:** Listed thermal conductivity of CFRPs with functionalization.

Composite	Fiber	Matrix	Modification Type	Processing Technique	Thermal Conductivity (with Functionalization) (W/mK)	Thermal Conductivity (with Functionalization) (W/mK)	References
CF/GO/epoxy	AS4 3K 8HS Carbon fiber	1.0 wt.% GO/Epon 828 epoxy resin and Epicure W curing agent	Matrix modified	Compression molding	0.68	0.72	[16]
PAI/CFs/rGO	rGO/PAN-based carbon fiber	Polyamide-imide	Fiber modified	Compression molding	0.27	0.53	[29]
CF/GNP-epoxy	T700 carbon fiber	5 wt.% GNP/MVR444 epoxy resin and MVR444H hardener	Matrix modified	Vacuum-assisted resin infusion	0.6	0.8	[68]
CF/GN-ester	T700 carbon fiber	Bisphenol A dicyanate ester	Matrix modified	Mold pressing	4.89	5.10	[54]
GO-CF/PAPI	Carbon fiber	Polyaryl polymethylene isocyanate	Fiber modified	Hydraulic pressing	0.18	0.23	[63]
CF/GO-epoxy	Zoltek Panex carbon fiber	GO/ER 5300 epoxy resin	Fiber modified	Vacuum-assisted resin infusion molding	0.83	0.87	[66]
GNPs-CF/epoxy	0.5 wt.% GNPs-carbon fiber	LY1564 epoxy resin and Aradur 3486 hardener	Fiber modified	Vacuum-assisted resin infusion	0.53	0.84	[55]

**Table 3:** Listed electrical conductivity of CFRPs with functionalization.

Composite	Fiber	Matrix	Modification Type	Processing Technique	Electrical Conductivity (without Functionalization) (S/m)	Electrical Conductivity (with Functionalization) (S/m)	References
CF/GO/epoxy	AS4 3K 8HS Carbon fiber	1.0 wt.% GO/Epon 828 epoxy resin and Epicure W curing agent	Matrix modified	Compression molding	5.65 × 10^−4^	1.31 × 10^−3^	[16]
CF/GO/epoxy	CJ20T carbon fiber	6002 epoxy resin and HARTCURE-10 curing agent	Matrix modified	Hand lay-up	4.5	6.2	[55]
GNPs/CF/epoxy	GNPs/PAN-based AS4 carbon fiber	Epon 828 epoxy resin	Fiber modified	Prepreg and hand lay-up	5.6	6.8	[47]
CF/GNP-epoxy	T700 carbon fiber	5 wt.% GNP/MVR444 epoxy resin and MVR444H hardener	Matrix modified	Vacuum-assisted resin infusion	0.3	0.60	[68]
CF/GN-ester	T700 carbon fiber	GN/Bisphenol-A dicyanate ester	Matrix modified	Mold pressing	0.89	1.4	[54]
GO/CF/EP	GO/PAN-based carbon fiber	TDE-85 epoxy resin	Fiber modified	Vacuum-assisted resin transfer molding	1.02	1.37	[78]
GNPs-CF/epoxy	T700G carbon fiber	Bisphenol A dicyanate epoxy resin	Fiber modified	Vacuum-assisted resin transfer molding	6.4	8.9	[56]
CF/GO-epoxy	Zoltek Panex carbon fiber	GO/ER 5300 epoxy resin	Fiber modified	Vacuum-assisted resin infusion molding	0.34	0.561	[67]

**Table 4:** Listed viscoelastic properties of CFRPs with functionalization.

Composite	Fiber	Matrix	Modification Type	Processing Technique	Viscoelastic Properties (without Functionalization)	Viscoelastic Properties (with Functionalization)	References
CF/GO/PI	IM7 8552 carbon fiber	Laser-induced GO array/Polyimide substrate	Matrix modified	Manual pressured roller	Storage modulus (E’)-109 GPa Loss modulus (E’’)-2.3 GPa T_g_-210 °C	Storage modulus (E’)-100 GPa Loss modulus (E’’)-3.5 GPa T_g_-240 °C	[12]
LBL 10 Go/CF/PP	Carbon fiber	Polypropylene	Fiber modified	Knotting-melting	Storage modulus (E’)-6.03 GPa Loss modulus (E’’)-452.3 MPa T_g_-8.12 °C	Storage modulus (E’)-7.09 GPa Loss modulus (E’’)-571.1 MPa T_g_-10.48 °C	[39]
rGO/CF/epoxy	0.2 wt.% GO/Plain weave carbon cloth	LAPOX C51 epoxy resin and lapox AH-428 hardener	Fiber modified	Vacuum-assisted resin transfer molding	Storage modulus (E’)-5327 GPa Loss modulus (E’’)-57 GPa T_g_-47 °C	Storage modulus (E’)-7525 GPa Loss modulus (E’’)-63 GPa T_g_-59 °C	[58]
CF/GO/epoxy	CJ20T carbon fiber	6002 epoxy resin and HARTCURE-10 curing agent	Epoxy modified	Hand lay-up	Storage modulus (E’)-135 GPa Loss modulus (E’’)-87 GPa T_g_-41 °C	Storage modulus (E’)-150 GPa Loss modulus (E’’)-100 GPa T_g_-54 °C	[55]
PEI-FGO/epoxy/CF	Plain weave carbon cloth	LAPOX C51 epoxy resin and lapox AH-428 hardener	Fiber modified	Vacuum-assisted resin transfer molding	Storage modulus (E’)-302 GPa Loss modulus (E’’)-34 GPa T_g_-43 °C	Storage modulus (E’)-410 GPa Loss modulus (E’’)-49 GPa T_g_-58 °C	[57]
Salinized GO-CF/epoxy	0.5 wt.% GO/T700S carbon fiber	HS5392 epoxy resin	Fiber modified	Resin transfer molding	Storage modulus (E’)-37 GPa T_g_-97 °C	Storage modulus (E’)-40 GPa T_g_-109 °C	[36]
GO-CF/epoxy	0.3 wt.% GO/T300 carbon fiber	LY556 epoxy resin and TETA hardener	Fiber modified	Hand lay-up	Storage modulus (E’)-27.9 GPa T_g_-108.7 °C	Storage modulus (E’)-32.5 GPa T_g_-123.9 °C	[65]
GO-CF/epoxy	0.5 wt.% GO/T700 carbon fiber	PPBES resin	Fiber modified	Solution impregnation	Storage modulus (E’)-34.3 GPa T_g_-187 °C	Storage modulus (E’)-44.5 GPa T_g_-250 °C	[78]
GO-CF/epoxy	1 wt.% GO/Carbon fiber	DER331 epoxy resin	Fiber modified	Mold pressing	Storage modulus (E’)-85 GPa T_g_-123 °C	Storage modulus (E’)-120 GPa T_g_-175 °C	[79]

**Table 5:** Comparison of CFRP and steel cable properties [89].

Property	Tensile Strength	Elastic Modulus	Elongation at Rupture	Density	Thermal Coefficient of Expansion	Poisson’s Ratio
Steel cable	1670 N/mm^2^	205,000 N/mm^2^	6.0%	7850 kg/m^3^	1.2 × 10^−5^ K^−1^	0.3
CFRP Cable	2700 N/mm^2^	160,000 N/mm^2^	1.6%	1600 kg/m^3^	0.2 × 10^−6^ K^−1^	0.3
